# Celebrating 30 years of ART in Latin America; and the 2018 report‡§

**DOI:** 10.5935/1518-0557.20210055

**Published:** 2021

**Authors:** Fernando Zegers-Hochschild, Javier A. Crosby, Carolina Musri, Maria do Carmo Borges de Souza, A. Gustavo Martínez, Adelino Amaral Silva, José María Mojarra, Diego Masoli, Natalia Posada, Latin American Network of Assisted Reproduction (REDLARA)

**Affiliations:** 1 Unit of Reproductive Medicine, Clínica Las Condes, Lo Fontecilla 441, Santiago, Chile; 2 Program of Ethics and Public Policies in Human Reproduction, Facultad de Medicina, Universidad Diego Portales, Ejercito 260, Santiago, Chile; 3 Fertipraxis, Av. das Américas, 4.666 Sls 312/313, Barra da Tijuca, Rio de Janeiro, Brazil; 4 Fertilis, Av. Fondo de la Legua 277 (B1609JEC), San Isidro, Provincia de Buenos Aires, Argentina; 5 Genesis-Centro de Assistência em Reprodução Humana, SHLS cj L, Aales Tes L331, Brasília DF 70390-907, Brazil; 6 Hospital CIMA Hermosillo, Paseo Río San Miguel 35, Col. Proyecto Rio Sonora C.P, Hermosillo Sonora 83280, Mexico; 7 INSER, Calle 12 No. 39-60, Sector El Poblado Medellín, Colombia; 8 Latin American Network of Assisted Reproduction (REDLARA), Plaza Independencia 811, Montevideo, Uruguay

**Keywords:** Latin American Registry of ART, 30 years' trend analysis, safety, efficacy and perinatal outcome

## Abstract

**Objective:**

What are the trends in patient characteristics, effectiveness and safety of assisted reproductive technology (ART) performed in Latin America over the past three decades, as well as the detailed outcomes of procedures initiated in 2018?.

**Design:**

Retrospective collection of multinational data including epidemiology and outcomes of ART performed between 1990 and 2018.

**Results:**

Over these 30 years we report 955,117 initiated cycles, 191,191 deliveries and 238,045 live births. In 1990, 66.5% of women were ≤34 years and 8.7% ≥40 years; in 2018, 26.4% of women were ≤34 years and 32.0% were ≥40 years. In 1990, 60.4% of transfers included ≥3 embryos, falling to 13.5% in 2018, and single embryo transfer (SET) increased from 13.8% to 30.4% between 1990 and 2018. Delivery rate per fresh transfer increased from approximately 17% in the 1990s to 25% in 2018, with a meaningful drop in high-order multiples, from 5-9% in the 1990s to 0.4% in 2018. This drop is associated with increasing use of frozen embryo transfer (FET) (57% in 2018) compared with 10% in 2000. In 2018, delivery rate in FET was 28.3%, reaching 31.2% in freeze-all cycles; and the cumulative live birth rate (fresh + FET) was 41.9%. Elective SET also increased, from 0.9% in 2010 to 10% in 2018. The delivery rate in elective SET (31.7%) was only 5.4% lower than elective double embryo transfer (DET) (37.1%); however, multiple births increased from 2.1% to 25.5% twins and 0.4% triplets in elective DET.

**Conclusions:**

The Latin American Registry of Assisted Reproduction (RLA) celebrates 30 years of voluntary reporting from a total of nearly 200 centres in 15 countries. This South-South Cooperation network has proven to be an efficient and safe system for technological transfer and regional growth.

## INTRODUCTION

In this report, we celebrate the 30^th^ anniversary of the Latin American Registry of Assisted Reproduction (RLA), which for the past 25 years has been part of the Latin American Network of Assisted Reproduction (REDLARA).

In 1990, for the first time, 19 centres from eight countries (Argentina, Brazil, Chile, Colombia, Ecuador, Mexico, Panama and Venezuela) voluntarily reported the outcomes of treatment with assisted reproductive technologies (ART) to a centralized multinational organization. The forms for data collection were adapted from those developed by the International Working Group of Registers in Assisted Reproduction (now the International Committee for Monitoring Assisted Reproductive Technologies (ICMART). Over the years, these forms have been modified many times in order to comply with regional interests and the incorporation of new technology. Initially, data were collected using printed forms, sent by fax, but between 1990 and 1995, software was developed that included internal controls to check for consistency of the data reported, and by the end of 1995 all data were collected electronically and entered directly online (www.redlara.com).

After 20 years of reporting summary data, in 2010 the RLA started to develop a cycle-based registry (case by case), becoming the only multinational registry of this kind. The software used was field tested in several institutions and regional workshops were carried out in order to facilitate its implementation, which commenced in 2011. Today, professionals from each of the 191 participating centres in 15 countries can access their data with a centre-specific passcode. Furthermore, representatives of each participating institution can access tables and figures generated automatically and containing detailed information gathered from their own centre and also, detailed information from the country they represent, which serves as external quality control. The incorporation of a cycle-based registry has proved very useful in understanding the subtleties involved in the evaluation of outcome. For example, live birth data from single embryo transfer (SET) can be very misleading if it is not stratified according to elective and non-elective SET. The same applies to the comparison of live births after frozen embryo transfer (FET) resulting from an unsuccessful fresh cycle as compared with freeze-all cycles where the best embryos are cryopreserved for delayed transfer. With this cycle-based registry, centres now have now a greater armamentarium to examine their strengths as well as their weaknesses.

Starting in 1996, an accreditation team consisting of a biologist and a clinician from a different country certifies all centres reporting to the registry. There are strict regulations, including professional degree of the personnel responsible for laboratory procedures, equipment and facilities, protocols for quality control, documentation of specific consent forms duly signed by patients; and the achievement of minimum standards of success that need to be accomplished before the data from a specific centre are included in the registry.

Yearly reports between 1990 and 2011 are available as booklets or PDFs (downloadable from www.redlara.com); since 2012, reports have been simultaneously published in RBM Online and JBRA Assisted Reproduction, the official journal of REDLARA.

This report provides a trend analysis of patient characteristics, modality of treatments and outcome of ART procedures performed in Latin America between 1990 and 2018, as well as some specific data on utilization, effectiveness and perinatal outcomes of treatments initiated in 2018 and babies born up to September 2019.

## MATERIAL AND METHOD

This analysis includes ART procedures started between 1990 and 2018 and babies born up to September 2019. The latest report, included in this manuscript, includes 191 centres in 15 countries reporting cycles initiated in 2018 (Supplementary Table 1). Data are available for fresh autologous cycles of IVF and intracytoplasmic sperm injection (ICSI); preimplantation genetic testing (PGT); FET; oocyte donation, including the transfer of fresh and frozen-thawed embryos; fertility preservation; and vitrified-warmed oocyte cycles, both autologous and heterologous (FTO).

This report includes longitudinal data from 1990 to 2018 as well as specific data on treatments started on 1 January 2018 and babies born up to September 2019. Data on pregnancy and perinatal outcomes are obtained from follow-up of cohorts treated during this period.

The terminology used by RLA refers to definitions implemented by ICMART and first published in 2006 ^(Zegers-Hochschild et al., 2006)^, followed later by 'The International Committee for Monitoring Assisted Reproductive Technology (ICMART) and the World Health Organization (WHO) Revised Glossary on ART Terminology, 2009' ^(Zegers-Hochschild et al., 2009a, 2009b)^, further translated into Spanish and Portuguese in compliance with WHO regulations. Since 2017, the RLA has adopted the new terminologies included in 'The International Glossary on Infertility and Fertility Care, 2017' ^(Zegers-Hochschild et al., 2017)^.

As mentioned before, all centres reporting to the registry are certified by an accreditation team and although the criteria used for centre certification have changed over the years, the general principles remain. As part of the accreditation programme, all participating institutions agree to have their data registered and published by the RLA. Therefore, no other consent forms are requested for the scientific disclosure of these data. The latest accreditation forms can be found at: https://redlara.com/acreditacao.asp.

Methods of data collection have experienced minimal changes since 2012 when the cycle-based registry was fully implemented and can be found in ^Zegers-Hochschild et al. (2020a;b)^. In previous years, summary data were available, which makes it difficult to examine trends on very sophisticated variables such as outcome of blastocyst transfers or elective transfers, which have only been available since 2012. However, the data set of these 30 years is of great value when analysing global trends in the demography of women treated, the number of embryos transferred and overall, the way ART has been practised in Latin America during the last three decades and the impact of incorporating new technologies.

To test for the effect of age, number of embryos transferred and, since 2012, the effect of elective transfers and state of embryo development at transfer on the delivery rate per embryo transfer, logistic regression analyses are conducted in fresh, FET and oocyte donation cycles. When appropriate, a Chi-squared test was used to analyse independence of categorical variables. *p*<0.05 was considered statistically significant.

**Figure 1:**
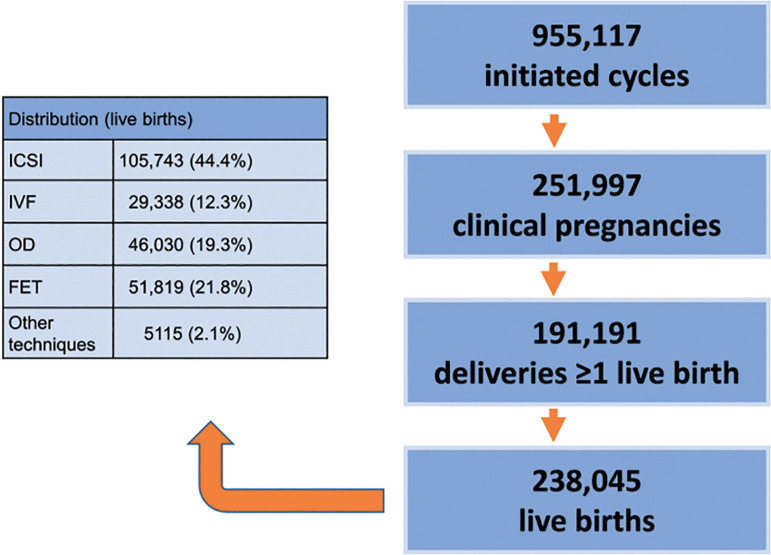
Database available at the Latin American Registry of Assisted Reproduction from January 1990 to September 2019.

The database for longitudinal analysis over these 30 years consists of 955,117 initiated cycles, 191,191 deliveries and the birth of 238,045 neonates (Figure 1), to which the three major contributors have been Brazil, Mexico and Argentina.

Because the cycle-based registry started in 2012, calculations of cumulative live births, the effect of blastocyst versus cleavage-stage embryo transfers and the effect of elective versus non-elective transfers can only be analysed from 2012 onwards.

## RESULTS

### A trend analysis from 1990 to 2018

Although different data collection systems have been used over these three decades, it is possible to examine changes in demographics as well as some of the characteristics that reflect the way ART has been practised throughout these years and how they have impacted the balance between safety and efficacy. Major changes in the age of female partners, number of embryos transferred, and the incorporation of new technologies, have all impacted women and the health of the children. The purpose of this longitudinal analysis is to understand how reproductive technology has evolved over time and its impact using standardized parameters to measure 'success', understood as the best possible equilibrium between efficacy, measured as the chances of achieving a live birth after a cycle is initiated or embryos are transferred; safety, measured primarily by the chances of avoiding multiple births, especially high-order multiples; and access as a measure of whether these technologies reach the majority of those in need.

#### Age of female partner and number of embryos transferred

**Figure 2:**
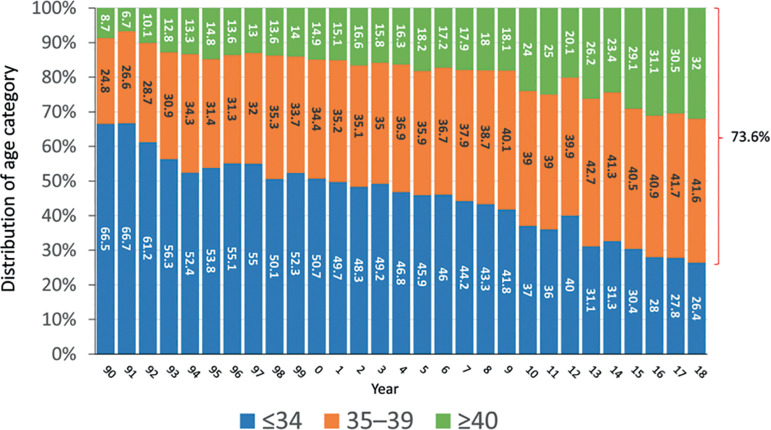
Age distribution of women for assisted reproductive technology (ART) cycles initiated between 1990 and 2018.

As seen in Figure 2, between 1990 and 2000, more than 50.0% of women were ≤34 years while only 14.9% were ≥40 years. In 2018, 32.0% of women treated were ≥40 years and only 26.4% were ≤34 years. This means that the proportion of women ≥35 increased from 49.0% in 2000 to 73.6% in 2018. With this change in demographics, it is difficult to compare the outcome of any treatment modality throughout time, unless the age of the female partner is standardized throughout the study period.

**Figure 3:**
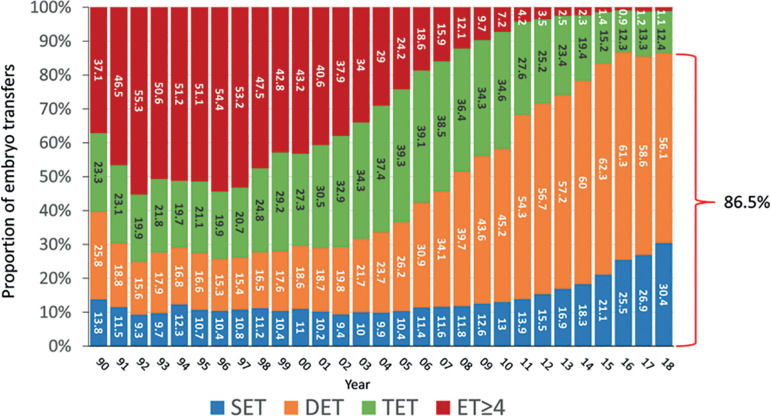
Distribution of embryos transferred in fresh IVF/intracytoplasmic sperm injection (ICSI) cycles between 1990 and 2018. DET = double embryo transfer; ET = embryo transfer; SET = single embryo transfer; TET = triple embryo transfer.

Similarly, when analysing the number of embryos transferred in fresh IVF and ICSI cycles (as seen in Figure 3), between 1990 and 2000, 60.4% to 75.2% of transfers include 3 and ≥4 embryos, dropping to 13.5% in 2018. Similarly, the proportion of SET increased from the lowest rate of 9.3% in 1992 to 30.4% in 2018.

#### Outcome of ART treatments and multiple births

**Figure 4:**
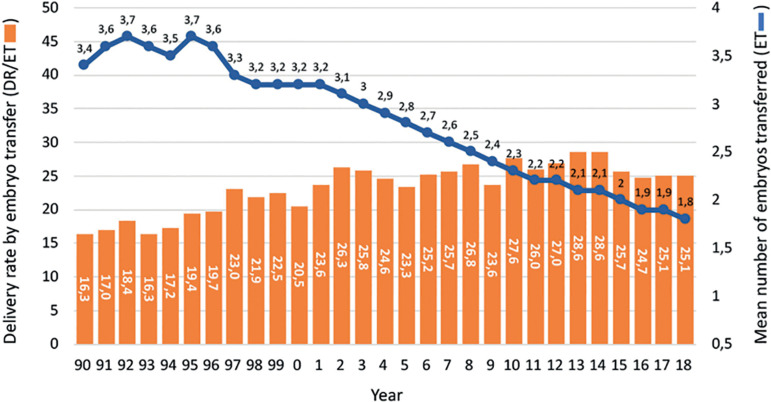
Delivery rate by fresh embryo transfer in Latin America between 1990 and 2018.

**Figure 5:**
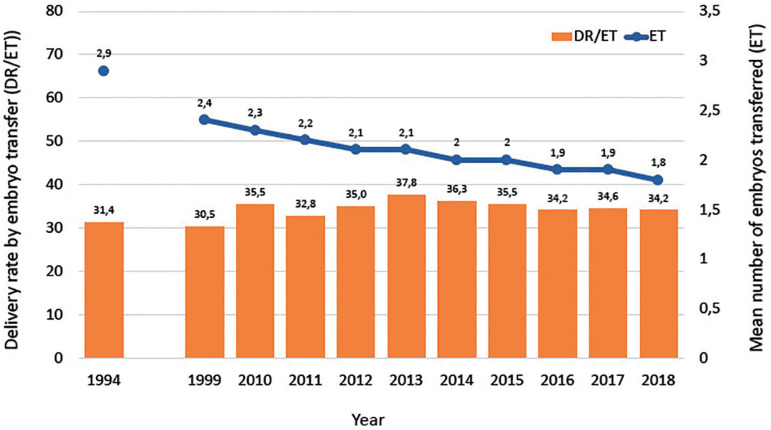
Delivery rate after fresh IVF/intracytoplasmic sperm injection (ICSI) in women <35 years between 1994 and 2018.

Although it might be disappointing, Figure 4 shows that in spite of the incorporation of vast amounts of frontline technology, the chances of delivering a live birth after a fresh transfer has increased by only 8.8% in almost three decades. However, what needs to be considered is that in the years 1990 to 1995, delivery rates per fresh embryo transfer of 16.3% to 19.4% were achieved in a population where only 6.7% to 14.8% of women were ≥40 years, while the vast majority (53.8% to 66.7%) of women were ≤34 years. Today, the proportion of women ≥40 has increased to 32% and only 26.4% of women are ≤34 years. Furthermore, between 1990 and 2000, the mean number of embryos transferred fluctuated between 3.2 and 3.7, while this number has dropped to a mean of 1.9 to 1.8 in recent years and the transfer of four embryos has dropped from more than 50% in the mid-1990s to 1% in 2018. A longitudinal analysis of birth rate after fresh embryo transfer in a selected population of women under 35 years (Figure 5) shows that the rise in birth rate does not exceed 3-4%. It is important to take into account that the higher delivery rate seen today results after a significant drop in the mean number of embryos transferred.

#### Number of embryos transferred and multiple births in fresh autologous cycles

**Figure 6:**
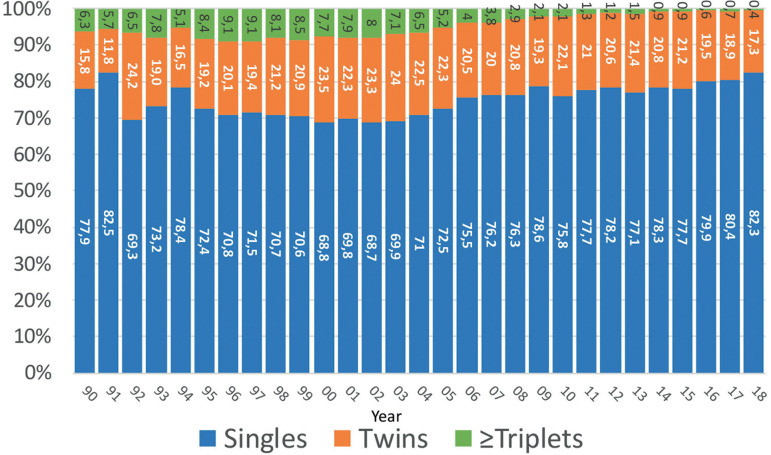
Proportion of births as singletons, twins and triplets or more between 1990 and 2018.

Figure 6 shows the impact of the number of embryos transferred on the proportion of twins and triplets and more. While in 2000 31.2% of deliveries were multiples, of which 7.7% were high order (triplets and more), in 2018 the proportion of multiple births dropped to 17.7%, of which triplets and more represent only 0.4%.

#### The impact of embryo cryopreservation on the outcome of ART

**Figure 7:**
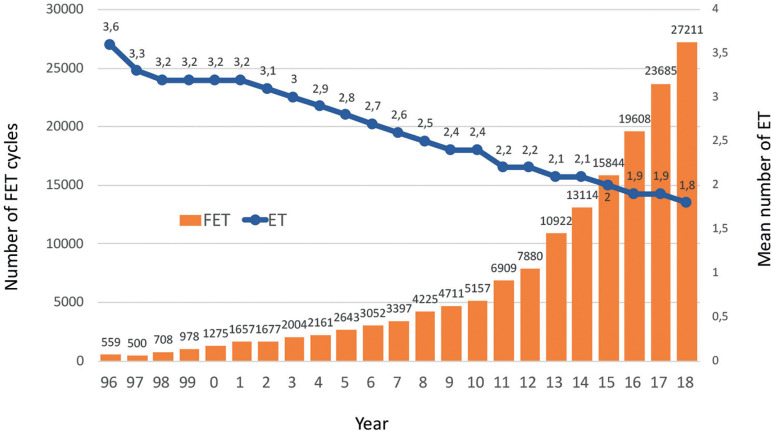
Number of frozen embryo transfer (FET) cycles and mean number of embryos per transfer in Latin America between 1996 and 2018.

**Figure 8:**
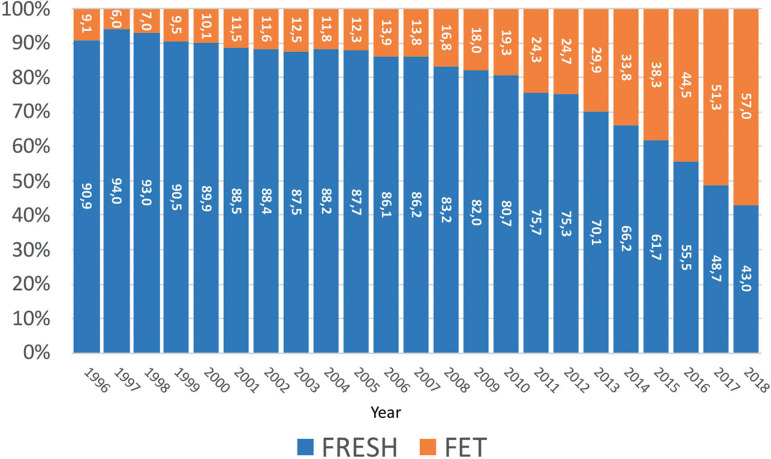
Proportion of FET and fresh transfers in Latin America between 1996 and 2018.

Much of the fall in the number of embryos transferred and in the proportion of multiple births has resulted from the incorporation of more efficient and safe methods to cryoprotect embryos, leading to an increased utilization of FET. Although FET was first reported in the RLA in 1994 as isolated events in cases of oocyte donation, its consistent use in global ART began in 1996 with almost 600 cases reported, which in 2018 included 27,211 initiated FET cycles (Figure 7). Furthermore, the proportion of FET to fresh cycles increased from 9% in 1996 to 57% of all transfers in 2018 (Figure 8).

**Figure 9:**
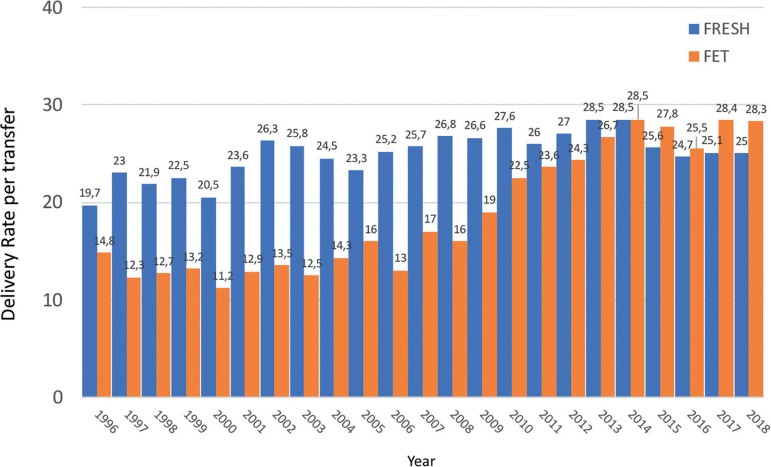
Delivery rate per embryo transfer in fresh and frozen embryo transfers in Latin America between 2000 and 2018.

A longitudinal analysis describing delivery rates of FET cycles as compared with fresh transfers can be seen in Figure 9. In the mid-1990s, embryo cryopreservation was considered a rescue procedure for supernumerary embryos, and in fact, up to 2008, the delivery rate with FET fluctuated between 12% and 17%. From then onwards, a steady rise in the use of FET has been accompanied by increasing success rates, reaching in 2018 a delivery rate by embryo transfer of 28.3%, which is 3.3% higher than the delivery rate of fresh transfers. So, when looking at overall success rates in these three decades, the increase in delivery rate is above 10-11% when comparing fresh transfers in the early 1990s and fresh + FET transfers in 2018.

There may be several reasons for the higher birth rate in FET over fresh transfers. Indeed, cryobiology has progressed over time and the incorporation of rapid vitrification and warming techniques in the mid-2000s has contributed to better quality embryos. Furthermore, between 2014 and 2018, the use of PGT has doubled from 14% to 28%; the proportion of freeze-all cycles has also increased from 32.9% to 40.6% of initiated cycles and the proportion of blastocyst transfers is also higher in FET than in fresh transfers. These three conditions contribute to selecting better quality embryos and are, at least in part, responsible for an increasing birth rate after FET over fresh transfers.

#### The influence of incorporating blastocyst transfer and elective transfer on ART outcomes

Blastocyst transfers were first systematically reported in 2000 and for the first 5 years represented 3-6% of all transfers, with clinical pregnancy rates by embryo transfer between 26% and 39%. In those years, however, 65-70% of transfers included ≥3 embryos, irrespective of the stage of embryo development at transfer. Between 2010 and 2018, the proportion of blastocyst transfers has steadily increased, reaching 43.0% of fresh IVF/ICSI transfers in 2018, with a delivery rate per transfer of 31.1%, compared with 21.0% when transferring cleaving embryos.

Given that the majority of embryos are cryopreserved at a blastocyst stage, most FET cycles are performed with blastocysts, which in part explains the higher birth rate after FET compared with fresh transfers (Figure 9).

**Figure 10:**
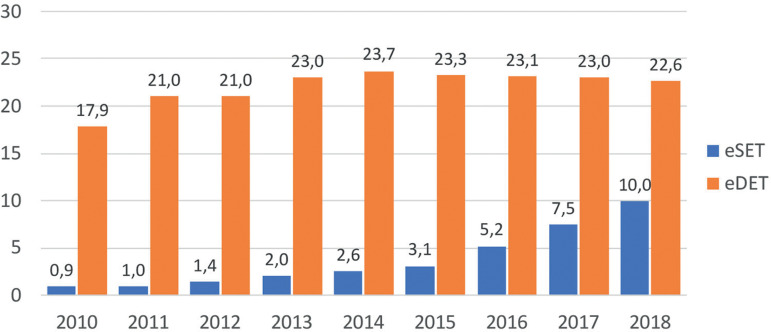
Proportion of elective single (eSET) and elective double (eDET) embryo transfers in Latin America between 2010 and 2018.

**Figure 11:**
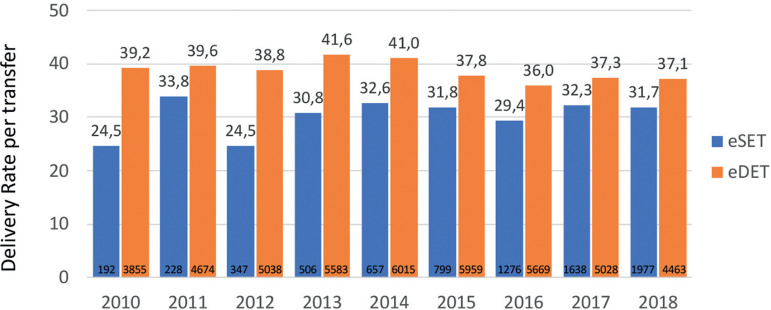
Delivery rate after elective single (eSET) and elective double (eDET) embryo transfers in Latin America between 2010 and 2018.

Elective single and elective double embryo transfers have also contributed to increasing birth rate in selected groups of women. Indeed, women having elective transfers are those with more embryos available for transfer and therefore represent an overall subpopulation of more fertile women. As seen in Figure 10, the use of elective SET and elective DET increased from 0.9% and 17.9% in 2010 to 10.0% and 22.6%, respectively, in 2018. Indeed, the actual proportion of elective SET in Latin America remains low, partly as a result of the absence of enforced national policies. It also results from the fact that in 2018, 73.6% of women were ≥35 years and 32% were ≥40 years; therefore, fewer women have large numbers of good embryos available for elective transfer. Interestingly, while in 2010 the rise in delivery rate obtained from elective DET over elective SET was almost 15%, in 2018 this difference dropped to only 5.4% (Figure 11). This minuscule rise in birth rate with elective DET over elective SET is accompanied by a dramatic rise in multiple births. In 2018, the proportion of twins and triplets increased from 2.1% of monozygotic twins with elective SET to 25.5% of twins and 0.4% of triplets after elective DET, as reported below.

#### Perinatal mortality and preterm births

The way ART is practised has a great impact on perinatal health as well as child development. As seen in Table 1, perinatal mortality, in a universe of 243,005 births with full biomedical data and collected over three decades, is twice as high in twins as in singletons (26.7‰ and 12.8 ‰, respectively) and 5.3 times higher in triplets and more (68.2‰ and 12.8‰, respectively). In a longitudinal analysis, overall perinatal mortality dropped from 32‰ in 1990 and 38.5‰ in 2000 to 11.6‰ in 2010 (www.redlara.com) and 14‰ in 2018 (Supplementary Table 2). Another marker that reflects the influence of ART practice in child health results from preterm birth, and especially, extremely preterm birth. The prevention of preterm and extremely preterm births has also experienced changes in these three decades. Overall preterm births fell from a range of 30-38% between 1990 and 2010 (www.redlara.com) to 26.6% in 2018. However, the most important consequence of decreasing the number of embryos transferred has been lowering the proportion of extremely preterm birth, from 28.8% of all preterm deliveries in the 1990s to 10.8% in 2000, and 1.7% in 2018 (Supplementary Table 3).

**Table 1.** Perinatal mortality according to gestational order from 1990 to 2018.

	Singleton	Twins	≥ Triplets
**Livebirth^a^**	144,637	79,755	13,562
**Stillbirth**	1168	1128	477
**Early neonatal death**	704	1058	516
**Perinatal Mortality^b^**	12.8‰	26.7‰	68.2‰

(a) Early neonatal death are excluded

(b) Perinatal Mortality = (stillbirth + early neonatal death) / (livebirth + stillbirth + early neonatal death)

### Characteristics and outcomes of ART procedures initiated in 2018 and births up to September 2019

#### Participation

A total of 191 centres in 15 countries reported 104,169 ART procedures initiated during 2018. This represents more than 70% of centres in the region. Most centres were located in Brazil (n=64), followed by Mexico (n=37) and Argentina (n=26) (Table 2). Compared with 2017, two centres, having stopped reporting, resumed their participation; seven centres either closed or stopped reporting and eight new centres were accredited by REDLARA and their data incorporated in 2018, contributing with 3396 out of 10,569 more cycles reported in 2018 with respect to the previous year. The mean number of initiated cycles by centre was 545.4, while 16.8% of centres reported more than 1000 initiated cycles; and the major contributors were in Brazil, followed by Mexico and Argentina.

**Table 2.** Assisted reproduction techniques reported in Latin America, 2018.

Country	Centres	FP	FRESH	FET	OD	FTO	Total
**Argentina**	26	858	9,279	4,735	6,418	446	21,736
**Bolivia**	3	3	434	37	278	26	778
**Brazil**	64	3,510	23,052	13,989	3184	1552	45,287
**Chile**	11	411	1,840	1,134	824	243	4,452
**Colombia**	14	121	1,502	815	668	102	3,208
**Ecuador**	7	20	717	363	365	70	1,535
**Guatemala**	2	22	205	113	128	5	473
**Mexico**	37	451	7,027	3,409	4,725	293	15,905
**Nicaragua**	1	1	97	28	17	2	145
**Panama**	3	51	502	274	177	14	1,018
**Paraguay**	1	22	102	99	35	12	270
**Peru**	14	1,175	2,025	1,804	1727	903	7,634
**Rep. Dominicana**	2	0	73	25	40	0	138
**Uruguay**	2	39	646	357	233	80	1,355
**Venezuela**	4	3	134	29	65	4	235
**Total n (%)**	**191**	**6,687**	**47,635** **(45.7)**	**27,211** **(26.1)**	**18,884** **(18.1)**	**3,752** **(3.6)**	**104,169**

FET=frozen autologous embryo transfer; FP=fertility preservation; FRESH=initiated fresh autologous IVF/ICSI cycles; FTO=includes embryo transfer cycles using autologous and donated vitrified-warmed oocytes; OD=transfer of fresh or frozen embryos due to oocyte donation.

Out of 104,169 initiated cycles, 47,635 corresponded to IVF/ICSI (45.7%); 27,211 corresponded to FET (26.1%); 18,884 to oocyte donation (18.1%); 6687 to fertility preservation (6.4%) and 3752 cycles were reported as FTO (3.6%) (Table 2).

**Figure 12:**
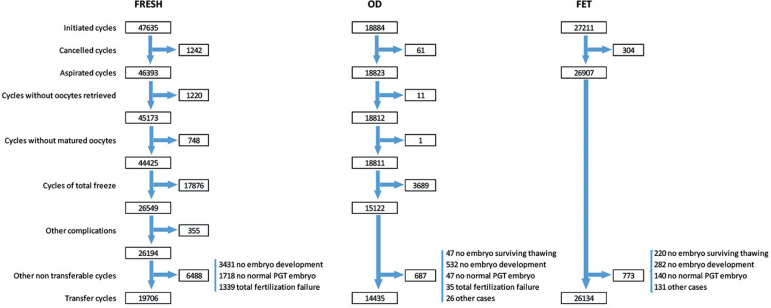
Number of cycles according to sequence of events that take place from the start of an assisted reproductive technology (ART) cycle until embryos are transferred in fresh, oocyte donation and frozen embryo transfer cycles in Latin America in 2018.

As described previously ^(Zegers-Hochschild et al., 2020a;b)^, a detailed description of the sequence of events that take place from the start of an ART cycle until embryos are transferred is described for 2018 in Figure 12. In cases of IVF/ICSI, there were only 19,706 embryo transfers out of 47,635 initiated cycles. Therefore, only 41.4% of initiated cycles were actually exposed to the chance of pregnancy, compared with 96.0% in FET and 76.4% of oocyte donation cycles. Reasons for discontinuation are important to consider when calculating outcome by initiated or aspirated cycle and when comparing outcomes in different techniques.

#### Utilization of ART in Latin America

**Figure 13:**
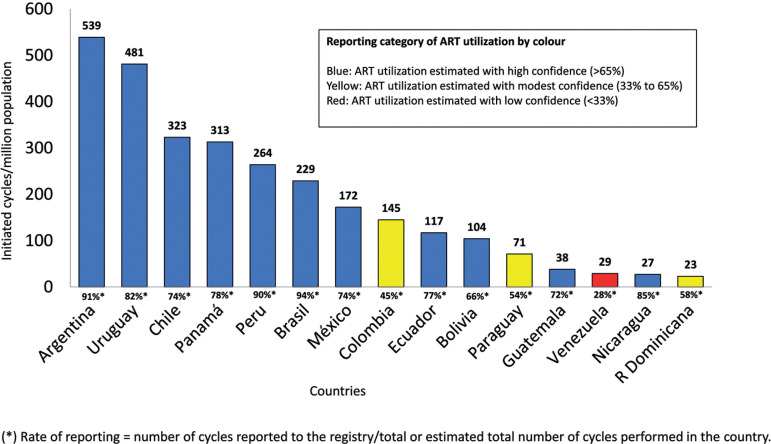
Utilization of assisted reproductive technology (ART) by country: estimated total number of cycles performed per million inhabitants in 2018.

Utilization of ART is expressed as the total number of cycles performed per million inhabitants. The way this has been calculated and estimated has been described previously ^(Zegers-Hochschild et al., 2020a^;b). Figure 13 represents an estimate of total number of cycles performed by each country. Given that the RLA collects between 70% and 90% of ART cycles in most countries, the estimate is fairly accurate, especially so with the major contributors in Latin America. Overall, Argentina and Uruguay, two countries with laws providing universal access to ART, have the highest utilization, with 539 and 481 cycles per million, respectively, followed by Chile, without laws but with recent public policies providing partial reimbursement, with 323 cycles per million. Brazil is the major contributor in the region, but its utilization is still poor compared with most European countries, with a mean utilization rate of 1400 cycles per million ^(European IVF-monitoring Consortium, 2020)^, very near the standard set by the ^ESHRE Capri Workshop Group (2001)^. Access to ART in Latin America has much room for improvement. Huge efforts have been made to stimulate countries to recognize the right to found a family as a human right. In 2012, the Inter-American Court of Human Rights, in an unprecedented ruling, obliged Costa Rica to restore IVF and make it available in the public health system (http://www.corteidh.or.cr/docs/casos/articulos/seriec257esp.pdf). Since then, several countries have been discussing reproductive rights as human rights; however, for the majority of Latin American countries, ART is still out of pocket funded.

### Outcome of pregnancies and deliveries

#### Fresh IVF and ICSI cycles

In 2018, 47,635 fresh IVF/ICSI cycles were initiated. After discarding aspirations without oocytes or absence of mature oocytes and excluding 17,876 cases of total embryo freezing and other factors (Figure 12), there were 19,706 embryo transfers, generating 6852 clinical pregnancies, with a clinical pregnancy rate of 25.8% per oocyte retrieval, and a delivery rate of 18.5% per oocyte retrieval and 25.0% per embryo transfer. Of these pregnancies, 89 were ectopic (1.3%), 16 induced abortions (0.23%) and 1237 ended in miscarriage (18.1%). A total of 587 pregnancies were lost to follow-up (8.57%) and 4923 deliveries were recorded. The clinical pregnancy and delivery rates in IVF and ICSI cycles are presented in Table 3. Of all fresh procedures, ICSI continues to dominate, representing 85.9%. Although there were no significant differences in the delivery rates per aspirated cycle, the difference per transfer was significantly higher in ICSI compared with IVF (25.4% and 22.8%, respectively; *p* = 0.0023; 95% confidence interval [CI] 0.95-4.21%).

**Table 3.** Clinical pregnancy rate and Delivery rate in FRESH autologous IVF/ICSI cycles in 2018.

ART procedure	Oocyte retrieval^a^	Clinical pregnancy rate per oocyte retrieval (n, %)	Delivery rate per oocyte retrieval (n, %)
**ICSI**	22,816	5,821 (25.5%)	4,214 (18.5%)
**IVF**	3,733	1,031 (27.6%)	709 (19%)
**Total**	26,549	6,852 (25.8%)	4,923 (18.5%)
***p*** **-value b**	---	0.0070	0.5754

ART=assisted reproductive technology; ICSI=intracytoplasmic sperm injection.

a Oocyte retrieval with at least one mature oocyte, excluding freeze-all cycles.

b IVF *versus* ICSI.

#### Oocyte donation cycles

**Figure 14:**
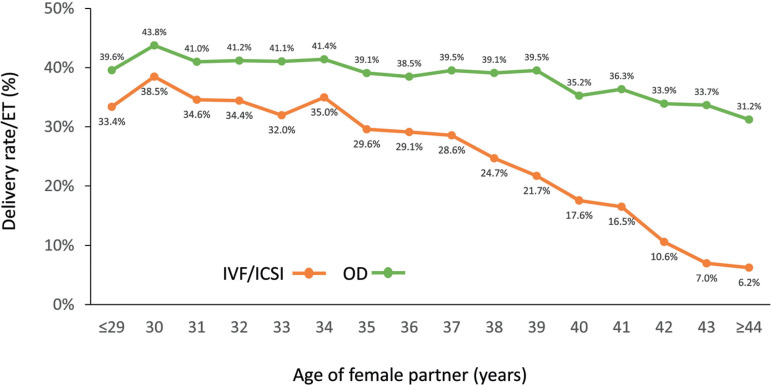
Delivery rate per embryo transfer (ET) in IVF/intracytoplasmic sperm injection (ICSI) and oocyte donation (OD) cycles according to the age of the female partner in Latin America in 2018.

As seen in Figure 12, in 2018, 18,884 oocyte donation cycles were initiated and, after removing freeze-all cycles of both oocytes and embryos, and cases without suitable embryos for transfer, there were 14,435 embryo transfers of both fresh and FET oocyte donation. As seen in Table 4, the clinical pregnancy and delivery rates per embryo transfer were significantly higher in fresh transfers than in FET (both *p*<0.0001). Furthermore, both clinical pregnancy and delivery rates after FET oocyte donation were higher than FET with autologous oocytes. Also, in contrast to autologous reproduction, the delivery rate after egg donation was only marginally affected by the age of the recipient (odds ratio [OR] 0.98; 95% CI 0.97-0.98). A significant drop in delivery rates compared with younger women is only seen after the recipient is ≥44 years old (*p*=0.001, 95% CI -3.06 to 9.49%) (Figure 14).

**Table 4.** Clinical pregnancy rate and delivery rate by embryo transfer in oocyte donation and FET cycles in 2018.

ART procedure	Embryo transfer	Clinical pregnancyper embryo transfer (n, %)	Delivery rateper embryo transfer (n, %)
Fresh oocyte donation	6903	3363 (48.7%)^a^	2388 (34.6%)^b^
Vitrified-warmed embryo transfer (oocyte donation)	7532	3158 (41.9%)^a^	2336 (31.0%)^b^
Vitrified-warmed embryo transfer (own)	26134	10328 (39.5%)	7398 (28.3%)

ART=assisted reproductive technology; CI=confidence interval; FET=frozen embryo transfer.

a (*p*<0.0001) 95% CI 5.17% to 8.43%

b (*p*<0.0001) 95% CI  2.06% to 5.14%

#### Frozen embryo transfer (FET)

In 2018, there were 27,211 FET, representing 26.1% of all procedures. This constitutes a rise of almost 15% compared with 2017. In relation to this, the overall mean number of embryos transferred (fresh + frozen) continues to drop, from 1.9 in 2017 to 1.8 (Figure 7). Of all initiated FET cycles, 1077 were cancelled or discontinued. Reasons for discontinuation were non-survival after warming, lack of chromosomally normal embryos, no embryo development or abnormal endometrium. After 26,134 completed FET cycles, the overall clinical pregnancy and delivery rates per transfer were 39.5% and 28.3%, respectively (Table 4), which is significantly higher than the clinical pregnancy and delivery rates after fresh transfers (*p*<0.0001). The higher clinical pregnancy and delivery rates in FET compared with fresh transfers are observed across all numbers of embryos transferred. The higher clinical pregnancy and delivery rates in FET over fresh transfers were especially evident in SET (Supplementary Tables 4 and 5).

#### Outcome of FET after total embryo freezing

A total of 17,876 cycles of total embryo freezing were reported, 21.7% more than in 2017. Of these, an average 3.75 embryos (SD 3.05) were cryopreserved and a mean of 1.6 (1 to 4) embryos transferred at a later stage. Aspirations followed by total embryo freezing gave rise to 7130 FET cycles resulting in 2225 births and a delivery rate per embryo transfer of 31.2%; this was higher than the delivery rate per embryo transfer of 28.3% in non-freeze-all FET (*p*<0.0001). A second FET attempt from embryos generated after a freeze-all cycle was reported in 1180 cases, with 316 subsequent deliveries. The delivery rate per embryo transfer in this attempt was 26.8%. Therefore, adding all transfers from this subset of total embryo freezing, the delivery rate per embryo transfer adds to 30.6%. The mean age of women was 35.5±4.6 years. When stratified by number of embryos transferred, the delivery rate per embryo transfer was 28.4% in SET and 35.3% in DET, respectively.

#### Number of embryos transferred, deliveries and multiple births after IVF/ICSI according to the age of women

In women ≤34 years, there were 5543 fresh transfers. The mean number of embryos transferred was 1.82 (range 1-5). In this age group, 28.2% were SET, of which 48.6% were elective SET. DET corresponded to 61.9% of transfers, of which 50.9% were elective DET. The transfer of three embryos and four or more embryos was carried out in 9.4% and 0.5% of cases.

In women of 35-39 years, there were 8669 fresh transfers. The mean number of embryos transferred was 1.86 (range 1-5). In this age group, 29.2% were SET, of which 36.7% were elective SET. DET corresponded to 56.2% of transfers and 41.2% were elective DET. The transfer of three embryos and four or more embryos were carried out in 14.1% and 0.4% of cases.

In women ≥40 years, there were 5494 fresh transfers. The mean number of embryos transferred was 1.85 (range 1-5). In this age group, 34.1% were SET, of which only 15.4% were elective SET, 49.2% were DET, 26.2% elective DET and 14.2% transfer of three embryos; the transfer of four or more embryos occurred in 2.6% of transfers.

The overall number of embryos transferred and multiple births after IVF/ICSI are presented in Supplementary Table 4. The mean number of embryos transferred was 1.85 (range 1-5). There were 5968 SET (30.3%) and 11,003 DET (55.8%), and 2735 transfers with three or more embryos took place (13.9%).

Overall, the clinical pregnancy and delivery rates per embryo transfer reached 34.8% and 25.0%, respectively. In terms of multiple births, of the 4923 IVF/ICSI deliveries registered, 82.7% were singletons, 16.7% were twins and 0.6% were triplets or more.

#### The influence of elective embryo transfer

Given that both SET and DET constitute heterogeneous groups, IVF and ICSI outcomes were further stratified after elective SET over oSET (only one embryo available for transfer) and elective DET over oDET (only two embryos available for transfer). As seen in Table 5, significant differences are observed in delivery rate per embryo transfer in both elective SET and elective DET over oSET and oDET (both *p*<0.0001); furthermore, the rate of twins and triplets increases with elective DET, whereas elective SET by itself does not seem to increase the rate of monozygotic twins. These data also show that when there are two good embryos for transfer, selecting one embryo (elective SET) has far better outcome compared with oDET, as the delivery rate is higher (31.7% versus 22.0%) and multiple birth rate drops from 16.6% in DET to 2.1% in elective SET. When there are ≥3 embryos for transfer, elective DET increases the chances of birth by only 5.4% over elective SET, but results in 25.9% of multiple births. These effects are even more pronounced in elective blastocyst transfer where multiple birth is almost 30% after elective DET compared with 2.4% of monozygotic twins after elective SET (Supplementary Table 6.

**Table 5.** Clinical pregnancy rate, delivery rate and gestational order in elective and non-elective SET and DET in fresh autologous IVF/ICSI in 2018.

Number of embryos transferreda	Total embryos transferred		Clinical pregnancies		Deliveries							
	n	%	n	%	No. of deliveries	Delivery rater per embryo transfer (%)^b^	Singleton (n)	Singleton (%)	Twin (n)	Twin (%)	≥Triplets (n)	≥Triplets (%)
**oSET**	3991	66.9	727	18.2	478	12.0	469	98.1	9	1.9	0	0.0
**eSET**	1977	33.1	842	42.6	627	31.7	614	97.9	13	2.1	0	0.0
**oDET**	6540	59.4	2044	31.3	1441	22.0	1196	83.0	239	16.6	6	0.4
**eDET**	4463	40.6	2207	49.5	1654	37.1	1226	74.1	422	25.5	6	0.4

DET=double embryo transfer; ICSI=intracytoplasmic sperm injection; SET=single embryo transfer.

a oSET or oDET: non-elective single or double embryo transfer; eSET or eDET: elective single or double embryo transfer.

b DR/ET: oSET and eSET *p*<0.0001; 95% CI 17.40-22.02%; oDET and eDET *p*<0.0001; 95% CI 13.35-16.85%.

#### Number of embryos transferred, deliveries and multiple births after oocyte donation and FET

Supplementary Tables 7a and 7b provide the clinical pregnancy and delivery rates according to the number of embryos transferred and multiple births in oocyte donation (fresh and FET). The mean number of embryos transferred in this group was 1.69 (range 1-5). In oocyte donation there were 6028 SET (2496 in fresh oocyte donation and 3532 in FET-oocyte donation), which correspond to 41.8% of embryo transfers. Of these, 1517 were elective SET (25.2% of SET), representing only 10.5% of all embryo transfers in oocyte donation. There were 6755 DET corresponding to 46.8% of embryo transfers. Of these, 1916 were elective DET, representing 13.3% of all transfers in oocyte donation. Overall, delivery rate per embryo transfer was 32.7%. Of the 4724 deliveries registered, 78.9% were singletons, 20.5% were twins and 0.6% were triplets and higher.

Supplementary Table 8 provides the clinical pregnancy and delivery rates according to the number of embryos transferred and multiple births in FET cycles. The mean number of embryos transferred was 1.62 (range 1-5). There were 11,743 SET (44.9%) and 12,788 DET (48.9%). Overall, the clinical pregnancy and delivery rates per embryo transfer reached 39.5% and 28.3%, respectively. Of the 7398 deliveries registered, 84.6% were singletons, 15.1% were twins and 0.3% were triplets and higher.

#### Influence of the stage of embryo development at transfer

Overall, 52.5% of embryo transfers were performed as blastocysts. The proportion of blastocyst transfers in FET (76.6%) was almost double the proportion in fresh IVF/ICSI (43.0%). This is important to consider when comparing outcomes between fresh and FET. In oocyte donation (both fresh and frozen), the proportion of blastocyst transfers reached 76.4%, which is 7.0% more than in 2017.

In fresh IVF/ICSI, the delivery rate after 8480 blastocyst transfers was 31.1% compared with 20.3% after the transfer of 11,209 cleaving embryos (*p*<0.0001). In oocyte donations, the delivery rate per embryo transfer was 34.7% in blastocyst transfers and 26.9% in cleaving embryo transfers (*p*<0.0001); and in FET, delivery rates per embryo transfer were 30.9% and 20.1%, respectively (*p*<0.0001). Blastocyst transfer was always associated with higher delivery rate compared with cleavage-stage embryos, irrespective of whether fresh or frozen, and the number of embryos transferred.

#### Preimplantation genetic testing (PGT)

In Latin America 140 out of 191 centres reported 8055 cycles where PGT was practised; 7303 cycles were fresh autologous (15.3% of oocyte retrievals) and 752 in oocyte donations (6.7% of retrievals). Overall, PGT was performed in 24,327 blastocysts (93.9%) and 1591 cleaving embryos (6.1%). In total, 10,264/25,918 blastocysts and cleaving embryos were euploid (39.6%).

In 2018, there were 3337 PGT transfer cycles of which 2694 were autologous (80.7%) and 643 from oocyte donation (19.3%). The mean age of women undergoing PGT with autologous eggs was 38.3 years (SD 4.3) and 25.5 years (SD 4.1) in egg donors.

In autologous cycles, the mean number of normal (euploid) embryos was 1.1 over a mean of 3.1 (SD 2.2) embryos biopsied. In oocyte donation cycles, the mean number of normal embryos was 2.8 over a mean of 4.5 (SD 4.9) biopsied. The delivery rate per embryo transfer in autologous cases was 30.9% and 33.7% in oocyte donation.

#### Effect of type of treatment on miscarriage

Globally, the rate of miscarriage in 6852 pregnancies resulting from autologous fresh embryo transfer was 18.1% compared with 16.8% miscarriages in 10,551 pregnancies after FET. When stratified by age, this difference is only significant in women ≥40 years, with 29.4% in fresh IVF/ICSI and 22.1% in FET (*p*<0.0001; 95% CI 4.16-10.48%). As expected, miscarriage rate in a total of 7522 pregnancies with donor oocytes was lower both in fresh transfers (13.7%) and in FET-oocyte donation (16.2%). Furthermore, in 1001 cases of oocyte donation using vitrified-warmed oocytes (FTO), the miscarriage rate was also lower (14.1%).

#### Effect of PGT on miscarriage

Globally, the rate of miscarriage in 1078 pregnancies using PGT reached 14.2% in pregnancies after FET. The effect of PGT on miscarriage varies according to the age of the female partner and is presented in Supplementary Table 9. When comparing miscarriage after autologous FET with and without PGT, the rate of miscarriage is significantly lower in women ≥40 years, from 23.5% to 16.0% (*p*=0.0032); as in women 35-39 years (17.0% to 13.2%; *p*=0.0355). In women younger than 35 years, PGT does not seem to decrease the chances of miscarriage.

#### Fertility preservation

A total of 6687 initiated cycles for fertility preservation were reported in 2018, representing a 27.2% increase over 2017. The mean age of women was 36.1 years (≤34 years 25.5%; 35-39 years 50.2%; and 40 years and above 24.3%). No oocytes were available for cryopreservation in 375 follicular aspirations (5.6%). The mean number of oocytes cryopreserved was 7.6, with large variations depending on the age of women (≤34 years 10.6; 35-39 years 7.2; and 4.8 in women ≥40 years). Reasons for fertility preservation included the desire to postpone pregnancy in 3793 cases (56.7%), whereas cancer-related factors were reported in 403 cases (6.0%); risk of premature ovarian insufficiency in 479 cases (7.2%), and 2012 cases (30.1%) were reported as 'other conditions/diseases potentially affecting ovarian reserve'. More than 10 oocytes were cryopreserved in only 28.2% of women expressing the desire to postpone fertility; 36.5% in women having cancer treatment; and, as expected, the proportion dropped to only 17.1% in women with risk of premature ovarian insufficiency.

#### Cumulative delivery rate per embryo transfer

**Figure 15:**
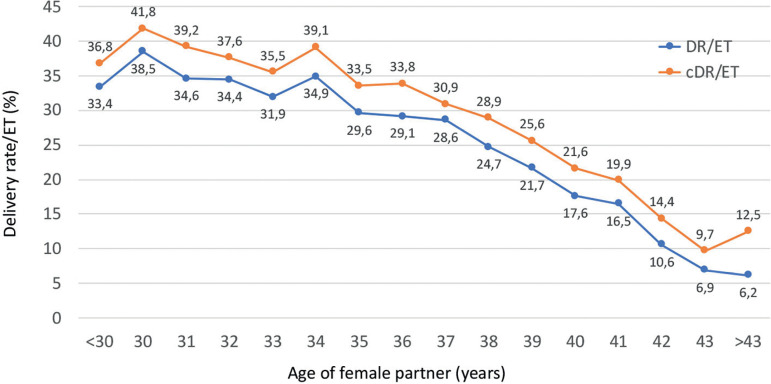
Delivery rate (DR) and cumulative delivery rate (cDR) per embryo transfer (ET) in IVF/intracytoplasmic sperm injection (ICSI) cycles according to the age of the female partner in Latin America in 2018.

Outcome of fresh embryo transfers and their consecutive FET were followed up in 9897 patients in 2018. This cohort included only women having surplus cryopreserved embryos resulting from their fresh transfer. Cohorts were followed until the first delivery after either fresh or vitrified-warmed transfers, or until all embryos were used. Taking all patients together, the delivery rate per embryo transfer increased from 25.0% after fresh embryo transfer to a cumulative rate of 41.9% (95% CI 15.98-17.82%; *p*<0.0001). The cumulative delivery rate per embryo transfer stratified by the age of the female partner at the time of oocyte retrieval is shown in Figure 15.

#### Perinatal outcome and complications

Perinatal mortality is presented in Supplementary Table 2. Data were available from 17,886 births and 21,137 babies born. The perinatal mortality increased from 9.2% births in 14,718 singletons, to 22.8% in 6174 twins and 93.9% in 245 triplets and higher. With 1044 more babies born than in 2017, multiparity increased perinatal death in similar proportion to previous years.

Gestational age at delivery was reported in 15,546 deliveries (86.9% of all deliveries). The mean gestational age at delivery was 37.73 (SD 2.2) weeks in singletons, 35.04 (SD 2.9) weeks in twins, and 32.15 (SD 2.8) weeks in triplets and higher. The overall risk of preterm birth (gestational weeks 22-36) increased from 17.4% in singletons, to 67.3% in twins, and 92.0% in triplets and higher. Furthermore, the risk of very preterm birth (gestational weeks 22-28) increased from 1.2% in singletons to 4.1% in twins and to 8.0% in triplets and higher (Supplementary Table 3). As reported in previous years, the mean weight of singletons born after FET (3151±569 g) was significantly higher than babies born after fresh transfer (3070±559 g) (*p*<0.0001); a similar relationship was seen after the birth of twins (2286±546 g after FET and 2236±542 g after fresh transfers; *p*=0.0053).

Complications during or directly derived from fresh procedures were reported in 244 out of 46,393 aspirations (0.53%). They include severe ovarian hyperstimulation syndrome, infections requiring antibiotics and vaginal or abdominal haemorrhage.

## DISCUSSION

In 1990, the Latin American Registry of Assisted Reproduction (RLA) published the first set of regional results, which included data collected from 19 centres in eight countries (www.redlara.com/registro.asp). This was the first regional initiative of this kind; 6 years later Australia and New Zealand made their first regional data set available online via the National Perinatal Epidemiology and Statistics Unit, since 2004 known as ANZARD. Furthermore, 10 years passed until a similar effort was reported in Europe by the European IVF Monitoring Consortium (EIM) as part of ESHRE ^(Nygren & Andersen, 2001)^. The RLA started as part of an initiative by the International Working Group of Registers in Assisted Reproduction, which later became the International Committee for Monitoring Assisted Reproductive Technologies (ICMART; www.icmartivf.org), in order to collect and publish a world report on ART. In fact, the forms used for data collection were initially adapted from those developed by the International Working Group.

In contrast to how the European registry was formed, in Latin America the multinational organization today known as REDLARA started as an ART registry, and it was only after 5 years of continuously publishing the regional registry, in 1995, that a group of embryologists and the clinical director from each of the 59 centres from 15 reporting countries gathered for the first time in Valparaiso, Chile, and decided to form the Latin American Network of Assisted Reproduction. Latin America was divided into five sub-regions with an elected regional director responsible for developing continuous educational programmes, hands-on working activities, and other sub-regional small group learning activities. Also, as part of REDLARA, an accreditation programme was initiated, which is until now, an independent body responsible for certifying the validity of the data reported, as well as certifying the existence of minimal standards of laboratory conditions and facilities, and the availability, quality and use of control programmes, infrastructure, equipment and personnel. Data from each centre are only accepted and incorporated in the RLA once the centre has been accredited. This programme has certainly evolved over time and the criteria used today in order to certify institutions can be found at: https://redlara.com/acreditacao.asp.

Today, REDLARA has become the largest regional organization, including more than 200 institutions in 15 countries. Furthermore, approximately 200 clinicians and embryologists have received their certification after completing a continuous education programme and a similar number are now participating in the programme. In this way, and differing from how the European registry was formed (as a mandate from ESHRE), in Latin America it was the registry that served as the backbone for the establishment of a regional network, as REDLARA.

The transfer of reproductive technology has been greatly facilitated by this regional network as part of what is referred to as South-South and Triangular Cooperation (https://www.unsouthsouth.org/about/about-sstc/). After the birth of Louise Brown in 1978, it took six and a half years for the birth of the first baby in Latin America. The reason it took so long has to do with the complexity of the technology required, and of course, generating human life in the laboratory was unthinkable in the vast majority of countries in Latin America. The positive impact of the RLA and REDLARA in the rapid transfer and dissemination of technology that followed has been quite remarkable. It has been within the umbrella of REDLARA that numerous hands-on workshops are organized sub-regionally, facilitating the movement and simultaneous training of biologists and clinicians among neighbouring countries. This south-south collaboration has generated strong regional bonding, which is an efficient way of sharing knowledge and experience.

ICSI: in 1992 the first publication of a birth after ICSI was communicated by ^Palermo et al. (1992)^; by 1993, 11 cases of ICSI were reported for the first time by the RLA, followed by more than 350 cases in the following year (www.redlara.com/registro.asp). ICSI rapidly became the most used form of fertilization in Latin American countries, today representing over 85% of fertilization procedures. It is worth noting that the Centers for Disease Control (CDC) in the USA only published their first results with ICSI in 1996; EIM did so in 1997.

Oocyte donation: another example of the rapid transfer and dissemination of technology in Latin America was the incorporation of oocyte donation, which was reported by the RLA for the first time in 1990. Forty cases were reported, of which 23 were the result of fresh embryo transfer, eight were frozen-thawed transfers and nine gamete intra-Fallopian transfer (GIFT). In the following 5 years, oocyte donation increased to 320 cases, and today, oocyte donation represents 18.1% of all ART procedures in the region (Table 2).

Frozen embryo transfer: FET was reported by the RLA as early as 1990, associated with oocyte donation, and for the first 5 years it was used mainly as a rescue procedure when surplus embryos were generated. It was only since 1996 that FET was formally reported as an independent procedure (Figure 7).

Reporting of more recent developments: the first 237 cases of PGT were reported by the RLA in 2005, preceded by a year by the EIM and followed by CDC in 2006. In 2018, there were 3337 PGT transfer cycles, of which 19.4% were performed in women <35 years, 39.2% in women 35-39 and 41.4% in women ≥40 years. Furthermore, 19.3% of PGT were performed in young oocyte donors, where 61.3% of embryos were euploid compared with only 36.3% in embryos generated from autologous reproduction. It is difficult to understand the need for PGT in properly selected young donors, but more and more, women and men seem to be unprepared to confront any form of uncertainty.

It was from 2012 onwards, with a fully implemented cycle-based registry, that it become possible to report the impact of new technologies as well as the follow-up of pregnancies resulting after incorporating new reproductive strategies. Such is the case with the reporting of the efficacy and safety of elective as compared with non-elective SET and DET (Table 5); and with the utilization of blastocyst transfer as compared with cleaving embryo transfers (Supplementary Table 6). Similarly, the perinatal outcome of pregnancies after FET or FTO can now be addressed in detail.

**Figure 16:**
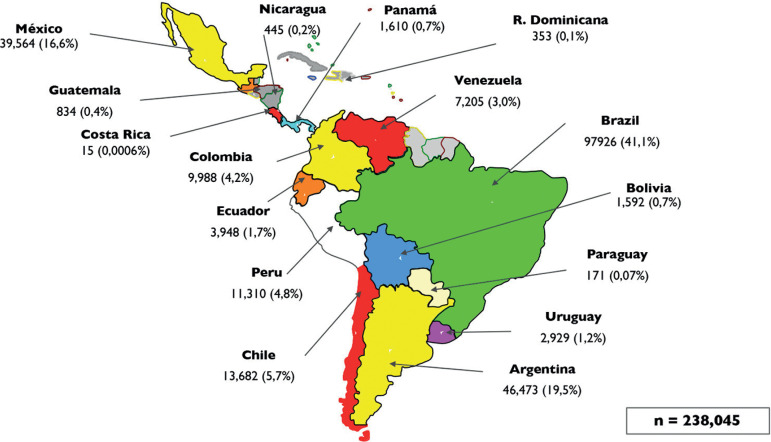
Number of live births by country reported to the Latin American Registry of Assisted Reproduction (RLA) between 1990 and September 2019.

The number of infants born from ART by country and the proportion of babies born from ART is most of all a reflection of access to treatment and specifically to the degree of ART utilization in that country. Figure 16 provides the total number of live births per country during these three decades, and Supplementary Table 10 provides the proportion of births from ART in relation to all births in 2018. Overall, the proportion of ART infants fluctuates between 0.04% of all births in Guatemala and Venezuela, and 0.9% of all births in Uruguay. The proportion of ART births by country follows a similar pattern to ART utilization by country (Figure 13). Thus, the proportion of ART births reflects utilization rather than quality of ART treatments. In countries like Denmark and Belgium, with ART utilization of more than 2000 cycles per million inhabitants, the proportion of ART infants is 5.1% and 4.6%, respectively ^(European IVF-monitoring Consortium, 2020)^; while in poorer countries with less coverage of ART, the proportion of infants born from IVF drops to less than 0.5%, both in Europe (Lithuania 0.1% and Serbia 0.2%) and in most of Latin America. The way we estimate the proportion of ART babies in Latin America follows the same principle used to estimate total number of initiated cycles per country. There is of course a source of error because the quality of centres not reporting to the RLA can be less efficient than centres that have been accredited by an independent body; and therefore, the assumption that the proportion of births per initiated cycle in those centres mimic reporting centres is a source of potential error. Nonetheless, irrespective of the magnitude of the error, the contribution of ART babies to the overall population is still very small in Latin America.

The United Nations refers to South-South Cooperation as a broad framework of collaboration between developing countries in the Global South (understood as countries with less developed social and economic conditions). It can take place on a bilateral, regional or inter-regional basis, its main purpose being to share knowledge, skills, resources and successful initiatives to meet development goals through concerted partnerships. This is what 19 institutions in eight countries voluntarily decided to accomplish 30 years ago.

As mentioned before, what started with the ART registry as the backbone for this South-South Cooperation has evolved into a sophisticated network of almost 200 centres from 15 countries which, year after year, not only voluntarily report their data, but open up their centres for external evaluation by an accreditation team, contribute to an ongoing education programme for clinicians and embryologists, facilitating hands-on training at certified institutions, and most importantly, share knowledge and experience with other institutions in the region, with the sole purpose of growing together in a respected cultural and ethnic identity.

Much of this South-South Cooperation has been facilitated by the transfer of technology, experience and knowledge from professionals 'in the North'. Latin America expresses its deepest gratitude to the late Professor Bob Edwards, who with no conditions or restraints, participated in numerous regional workshops in Mexico, Argentina, Brazil and Chile, among other countries, sharing knowledge and enthusiasm. Professor Richard Rawlings from the American Board of Bioanalysis (ABB) and Professor Klaus Wiemer who, for many years, visited many centres in the region to assist in the development of quality control programmes, contributing to our accreditation programme and helping with hands-on workshops for ICSI in the early 1990s, later for PGT and in the introduction of other techniques. This Triangular North-South Cooperation has been built upon friendship and care and has proved fundamental in the transfer of technology and in the building of strong and long-lasting cooperation.

South-South Cooperation between developing countries extends beyond a regional identity. For the past 10 years REDLARA, together with ANARA, the African Network and Registry of Assisted Reproduction, have established an intercontinental form of South-South Cooperation, again having the ART registry as a backbone ^(Dyer et al., 2020)^. Africa has built a collaborative network under a similar premise to REDLARA and today, all modifications in the software shared by both continents are discussed together before they are implemented, with enormous benefits to more than 30 countries in Latin America and Africa.

The authors express their deepest gratitude to all the centres that voluntarily contribute year after year to the RLA, and especially to the 19 centres that started this registry 30 years ago (www.redlara.com/registro.asp), most of which are still active and reporting. None of this effort would have been possible without the generous support of the pharmaceutical industry, Serono and Organon, for the first 15 years and today, by Ferring Pharmaceuticals.

REDLARA is probably the largest south-south health collaboration programme in Latin America. Today, together with ANARA, our sister organization in Africa, we have learned that the transfer of technology, often derived from the North, is best implemented, disseminated and made sustainable through regional organizations that provide institutions with a sense of belonging, reinforcing cultural and ethnic identity.

## Appendix

**Supplementary Table 1.** Centers and countries reporting to the Latin American Registry of Assisted Reproductive Technology, 2018.

CENTERS AND COUNTRIES REPORTING TO THE LATIN AMERICAN REGISTRY OF ASSISTED REPRODUCTIVE TECHNOLOGY, 2018	
ARGENTINA	
•	Servicio de Medicina Reproductiva, Instituto Gamma
•	Instituto de Fertilidad Asistida
•	Centro de Estudios en Ginecología y Reproducción (CEGYR)
•	Centro de Salud Reproductiva (CER)
•	Centro Integral de Ginecología, Obstetricia y Reproducción (CIGOR)
•	Centro de Investigaciones en Medicina Reproductiva (CIMER)
•	Centro de Medicina Reproductiva Bariloche , Fertility Patagonia
•	Centro de Estudios en Reproducción y Procedimientos de Fertilización Asistida (CRECER)
•	FECUNDITAS
•	FERTILAB
•	GESTAR
•	Centro de Reproducción Fertilequip
•	Fertya
•	FECUNDART
•	Centro de Reproducción, servicio de Ginecología Hospital Italiano
•	Mater, Medicina Reproductiva
•	Nascentis, Medicina Reproductiva
•	HALITUS, Instituto Médico
•	Instituto Medico de ginecología y Fertilidad PREFER
•	PREGNA, Medicina Reproductiva
•	Programa de asistencia reproductiva PROAR
•	PROCREARTE
•	Fertilidad San Isidro
•	SARESA, Salud reproductiva Salta
•	SEREMAS
•	VITAE, Medicina Reproductiva

BOLIVIA	
•	CENALFES
•	Instituto de Salud Reproductiva (ISARE)
•	EMBRIOVID, centro integral de reproducción y especialidades médicas
	
BRAZIL	
•	ANDROLAB, Clinica e Laboratório de Reprodução Humana e Andrologia
•	ANDROFERT, Centro de Referência em Reprodução Masculina
•	FERTIVITRO, Centro de Reprodução Humana
•	BIOS, Centro de Medicina Reprodutiva
•	FIV-MED
•	Clínica Geare
•	VIDA, Centro de Fertilidade
•	Clínica FERTWAY
•	NASCER, medicina Reprodutiva Ltda.
•	ORIGINARE, Centro de Reprodução Humana
•	CLINIFERT, Centro de Reprodução Humana
•	CONCEPTUS, Centro de Reprodução Assistida de Ceara
•	CONCEBER, Centro de Reprodução Humana
•	Clínica Origen
•	Clínica Pro-Genesis
•	Centro de Reprodução Humana CONCEPTION
•	Centro de Reprodução Humana MONTELEONE
•	Fértile Diagnósticos
•	CEERH, Centro especializado em Reprodução Humana
•	Embrios, Centro de Reprodução Humana
•	EMBRYOLIFE, Instituto de Medicina Reprodutiva
•	CENAFERT, Centro de Medicina Reprodutiva
•	Instituto VERHUM
•	Clínica FERTIBABY BH
•	Fertilcare, Centro de Reprodução Humana Ltda.
•	FECUNDA, Reprodução Humana
•	FELICCITA, Instituto de Fertilidade Ltda.
•	HUMANA, Medicina Reprodutiva
•	FERTILITY, Centro de Fertilização Assistida de Campo Grande
•	FERTILITY, Centro de Fertilização Assistida
•	FERTIL Reprodução Humana
•	REPROFERTY
•	FERTICLIN, Clínica de Fertilidade Humana
•	GENESIS, Centro de Assistência em Reprodução Humana
•	Genics, medicina Reprodutiva e Genômica
•	FERTIPRAXIS
•	GERA, Grupo de endoscopia e Reprodução Assistida
•	Clinica GERAR VIDA
•	Cegonha Medicina Reprodutiva
•	PRIMORDIA, Medicina Reprodutiva
•	Hospital de Clínicas de Ribeirão Preto
•	HUNTINGTON Campinas
•	HUNTINGTON, Centro de Medicina Reprodutiva (São Paulo)
•	JULES WHITE, Centro de Medicina Reprodutiva
•	HUNTINGTON Vila Mariana
•	Ideia Fertil, Santo André
•	Ideia Fertil, São Paulo
•	IMR, Instituto de Medicina Reprodutiva e Fetal
•	Insemine, Centro de Reprodução Humana
•	Centro de Reprodução Humana Santa Joana
•	Life Reprodução humana
•	FERTILITAT, Centro de Medicina Reprodutiva
•	Clínica MATRIX
•	Clínica Nidus
•	Centro de Reprodução Humana Nilo Frantz
•	Origen, Centro de Medicina Reprodutiva BH
•	Procriar, Centro de Medicina Reprodutiva e diagnósticos Ltda., Blumenau
•	Clínica PRO-CRIAR, Medicina Reprodutiva BH
•	Clínica PRO NASCER
•	Clínica ProSer
•	Centro de Reprodução Humana de São José do Rio Preto
•	GENESIS, Centro de Reprodução Humana
•	Centro de Reprodução Humana Prof. Franco Junior
•	Centro de Ensino e Pesquisa m Reprodução Assistida (CEPRA)
	
CHILE	
•	UMR Clínica de la Mujer Antofagasta
•	Centro de Estudios Reproductivos (CER)
•	Unidad de Medicina Reproductiva, Clínica Alemana
•	Unidad de Medicina Reproductiva, Clínica las Condes
•	Unidad de Medicina Reproductiva, Clínica de la Mujer
•	UMR clínica Indisa
•	Programa e Fertilización Asistida I.D.I.M.I.
•	Clínica Monteblanco
•	Centro de Fertilidad y Medicina Reproductiva Concepción S.A.
•	Centro de reproducción humana, Valparaiso
•	SG Fertility Chile
	
COLOMBIA	
•	Centro FECUNDAR, Cali
•	Unidad de fertilidad del Coutry ltda. CONCEPTUM
•	Centro de fertilidad Clinica de la mujer
•	Clinica Eugin
•	Asociados en Fertilidad y Reproducción Humana
•	FERTIVIDA
•	Clinica Machicado SAS
•	Centro Médico IMBANACO
•	Instituto de Fertilidad Humana S.A.S. (INSER Bogotá)
•	IN SER, Instituto Antioqueño de Reproducción (Medellín)
•	Procrear
•	Profamilia Fertil
•	Unidad de Fertilidad, Procreación Medicamente Asistida
•	Union temporal IN SER eje cafetero (Pereira)
	
ECUADOR	
•	Clínica de Medicina Reproductiva BIOGEPA
•	Centro Ecuatoriano de reproducción humana
•	Clínica INFES
•	Instituto Nacional de Investigación de Fertilidad y Esterilidad (INNAIFEST)
•	Instituto de Reproducción Humana Guayaquil
•	CONCEBIR, Unidad de Fertilidad y Esterilidad
•	Unidad de Fertilidad Hospital Alcívar
	
GUATEMALA	
•	Centro de Reproducción Humana S.A. (CER)
•	Centro Clinico Gestar (nuevo)
	
MEXICO	
•	Centro de Diagnóstico Ginecológico
•	Biofertility Center
•	Clinica Cerh S e RL de CV
•	URA, Unidad de reproducción asistida de Hispital CIMA Hermosillo
•	Centro de Cirugía Reproductiva y Ginecología, Unidad de Fertilización In Vitro (REPROGYN)
•	Instituto de Innovación Tecnológica y Medicina Reproductiva CITMER
•	Instituto para el estudio de la Concepción Humana IECH
•	Centro de Reproducción Asistida del Hospital Español (HISPAREP)
•	Centro de Reproducción Asistida del Occidente
•	Centro de Reproducción Asistida de Saltillo
•	Centro Universitario de Medicina Reproductiva
•	Fertility Center Cancún
•	Centro de Medicina reproductiva Filius
•	Genesis Centro de Fertilidad (Culiacan)
•	Ginecología y Reproducción Asistida GYRA
•	Unidad de Medicina Reproductiva del Hospital Angeles del Pedregal
•	IECH de Baja California
•	Instituto Mexicano de Alta Tecnología Reproductiva S.C. (INMATER)
•	Instituto de medicina reproductiva del Bajío IMER, sede Guadalajara
•	Concibo
•	Instituto Médico de la mujer (RED CREA)
•	Iinstituto VIDA Guadalajara-Instituto de Ciencias en Reproducción Humana
•	Instituto de Ciencias en Reproducción Humana, VIDA sede Matamoros
•	Centro especializado para la atención de la mujer (CEPAM)
•	INGENES DF
•	INGENES Guadalajara
•	Ingenes Monterrey
•	Instituto de Ciencias en Reproducción Humana (VIDA), sede León
•	Instituto de ciencias en reproducción humana del Sureste (Vida Merida)
•	Clinica Nascere
•	Plenus, Reproducción Asistida
•	PROGEN, Reproducción asistida y medicina fetal
•	Clinica de Infertilidad y reproducción asistida de Toluca SA de CV
•	Centro especializado en esterilidad y Reproducción Humana (CEERH)
•	Instituto de Ciencias en reproducción humana VIDA, ciudad de Mexico.
•	Centro CARE
•	Vida, Instituto de Reproducción Humana del Noroeste, Tijuana
	
NICARAGUA	
•	Centro de Fertilidad de Nicaragua
	
PANAMA	
•	IVI Panamá S.A.
•	Centro de reproducción Punta Pacífica
•	Instituto de salud femenina
	
PARAGUAY	
•	Neolife, Medicina y cirugía reproductiva
	
PERU	
•	Clínica CEFRA, Centro de Fertilidad y Reproducción Asistida
•	CERFEGIN
•	Centro de Fertilidad y Ginecología del Sur (CFGS)
•	Clinica de fertilidad del norte, Clinifer de Chiclayo
•	Centro de Fertilidad Germinar
•	FERTILAB
•	Inmater, Clinica de fertilidad
•	Instituto de Reproducción de la Clinica Ricardo Palma
•	Clinica Miraflores
•	Nacer
•	NiuVida
•	Grupo Pranor San Isidro, Clínica CONCEBIR
•	Grupo Pranor, Instituto de Ginecología y Reproducción Monterrico
•	Pranor, laboratorio de medicina reproductiva sede trujillo
	
REPUBLICA DOMINICANA	
•	Instituto de reproducción y ginecología del Cibao (IREGCI)
•	PROFERT
	
URUGUAY	
•	Centro de Esterilidad Montevideo (CEM)
•	Centro de Reproducción Humana del Interior
	
VENEZUELA	
•	FERTILAB
•	Unidad de Fertilidad, UNIFERTES
•	Instituto Venezolano de Fertilidad
•	Laboratorios In Vitro de Venezuela

**Supplementary Table 2.** Perinatal mortality according to gestational order in 2018.

	Singleton	Twin	≥ Triplets
**Livebirth***	14582	6033	222
**Stillbirth**	42	37	10
**Early neonatal death**	94	104	13
**Perinatal Mortality****	9.2%	22.8%	93.9%

(*) Early neonatal death are excluded

(**) Perinatal Mortality = (stillbirth + early neonatal death) / (livebirth + stillbirth + early neonatal death)

**Supplementary Table 3.** Gestational age and weight at birth according to gestational order in 2018.

Gestational Age (weeks) or Weight (gr)	Single		Twin		≥Triplets	
	**n**	**%**	**n**	**%**	**n**	**%**
22 - 28 weeks	150	1.2	114	4.1	6	8.0
29 - 36 weeks	2060	16.2	1741	63.2	63	84.0
≥37 weeks	10504	82.6	903	32.7	6	8.0
< 1000 gr	82	0.7	136	2.6	16	7.4
1000 - 2500 gr	1599	12.9	3383	65.0	195	89.8
> 2500 gr	10742	86.4	1689	32.4	6	2.8

**Supplementary Table 4.** Clinical pregnancy rate, delivery rate and gestational order according to the number of embryos transferred in fresh autologous IVF/ICSI in 2018.

Number of transferred embryos	Embryo transfers		Clinical pregnancies		Deliveries							
	Number	%	Number	CP (%)	Number of deliveries	Delivery rater per embryo transfer (%)	Singleton (n)	Singleton (%)	Twin (n)	Twin (%)	≥ Triplets (n)	≥ Triplets (%)
**1**	5968	30.3	1570	26.3	1105	18.5	1083	98.1	22	1.99	0	0.0
**2**	11003	55.8	4251	38.6	3095	28.1	2422	78.3	661	21.4	12	0.4
**≥3**	2735	13.9	1031	37.7	723	26.4	567	78.4	140	19.4	16	2.2
**Total**	19706	100.0	6852	34.8	4923	25.0	4072	82.7	823	16.7	28	0.6

**Supplementary Table 5.** Clinical pregnancy rate, delivery rate and gestational order according to the number of embryos transferred in Autologous FET in 2018.

Number of embryos transferred	Embryo transfers		Clinical pregnancies		Deliveries							
	Number	%	Number	CPR (%)	Number of deliveries	Delivery rater per embryo transfer (%)	Singleton (n)	Singleton (%)	Twin (n)	Twin (%)	≥ Triplets (n)	≥ Triplets (%)
**1**	11743	44.9	4177	35.6	2999	25.5	2945	98.2	54	1.8	0	0.0
**2**	12788	48.9	5521	43.2	3941	30.8	2971	75.4	961	24.4	9	0.2
**≥3**	1603	6.1	630	39.3	458	28.6	341	74.5	104	22.7	13	2.8
**Total**	26134	100.0	10328	39.5	7398	28.3	6257	84.6	1119	15.1	22	0.3

**Supplementary Table 6.** Clinical pregnancy rate, delivery rate and gestational order in elective and non-elective SET and DET in fresh autologous blastocyst IVF/ICSI in 2018.

Number of embryos transferred *	Embryo transfers		Clinical pregnancies		Deliveries							
	Number	%	Number	%	Number of deliveries	Delivery rater per embryo transfer (%) **	Singleton (n)	Singleton (%)	Twin (n)	Twin (%)	≥ Triplets (%)	≥ Triplets (n)
**oSET**	1423	46.7	370	26.0	247	17.4	240	97.2	7	2.8	0	0.0
**eSET**	1624	53.3	731	45.0	551	33.9	538	97.6	13	2.4	0	0.0
**oDET**	2293	49.4	865	37.7	631	27.5	487	77.2	141	22.3	3	0.5
**eDET**	2352	50.6	1287	54.7	956	40.6	671	70.2	281	29.4	4	0.4

**Supplementary Table 7a.** Clinical pregnancy rate, delivery rate and gestational order according to the number of embryos transferred in Fresh OD in 2018.

Number of embryos transferred	Embryo transfers		Clinical pregnancies		Deliveries							
	Number	%	Number	CPR (%)	Number of deliveries	Delivery rater per embryo transfer (%)	Singleton (n)	Singleton (%)	Twin (n)	Twin (%)	≥ Triplets (n)	≥ Triplets (%)
**1**	2496	36.2	1060	42.5	677	27.1	666	98.4	11	1.6	0	0.0
**2**	3405	49.3	1746	51.3	1291	37.9	906	70.2	382	29.6	3	0.2
**≥3**	1002	14.5	557	55.6	420	41.9	262	62.4	147	35.0	11	2.6
**Total**	6903	100.0	3363	48.7	2388	34.6	1834	76.8	540	22.6	14	0.6

**Supplementary Table 7b.** Clinical pregnancy rate, delivery rate and gestational order according to the number of embryos transferred in Frozen/thawed OD in 2018.

Number of embryos transferred	Embryo transfers		Clinical pregnancies		Deliveries							
	Number	%	Number	CPR (%)	Number of deliveries	Delivery rater per embryo transfer (%)	Singleton (n)	Singleton (%)	Twin (n)	Twin (%)	≥ Triplets (n)	≥ Triplets (%)
**1**	3532	46.9	1305	36.9	939	26.6	918	97.8	21	2.2	0	0.0
**2**	3350	44.5	1538	45.9	1147	34.2	829	72.3	314	27.4	4	0.3
**≥3**	650	8.6	315	48.5	250	38.5	147	58.8	93	37.2	10	4.0
**Total**	7532	100.0	3158	41.9	2336	31.0	1894	81.1	428	18.3	14	0.6

**Supplementary Table 8.** Clinical pregnancy rate, delivery rate and gestational order according to the number of embryos transferred in Autologous FET in 2018.

Number of embryos transferred	Embryo transfers		Clinical pregnancies		Deliveries							
	Number	%	Number	CPR (%)	Number of deliveries	Delivery rater per embryo transfer (%)	Singleton (n)	Singleton (%)	Twin (n)	Twin (%)	≥ Triplets (n)	≥ Triplets (%)
**1**	11743	44.9	4177	35.6	2999	25.5	2945	98.2	54	1.8	0	0.0
**2**	12788	48.9	5521	43.2	3941	30.8	2971	75.4	961	24.4	9	0.2
**≥3**	1603	6.2	630	39.3	458	28.6	341	74.5	104	22.7	13	2.8
**Total**	26134	100.0	10328	39.5	7398	28.3	6257	84.6	1119	15.1	22	0.3

**Supplementary Table 9.** Effect of PGT on miscarriage rate after FET in different age groups.

	FET with PGT	FET without PGT	
<35	33/238 13.9%	514/3559 14.4%	*p*=0.9065 95% CI -4.68% to 4.8%
35 to 39	67/508 13.2%	658/3876 17.0%	*p*=0.0355 95% CI 0.34% to 6.87%
>39	53/332 16.0%	426/1815 23.5%	*p*=0.0032 95% CI 2.7% to 11.78%

**Supplementary Table 10.** Number of births by country and proportion of birth from ART, Latin America 2018.

Country	Total number of births	Births registered by RLA	Estimated total number of births from ART	Estimated proportion of birth from ART
Argentina	685.394	3.810	4.187	0.61
Bolivia	233.722	204	309	0.13
Brazil	2.915.410	7.934	8.440	0.29
Chile	221.731	1.086	1.468	0.66
Colombia	649.742	891	1.980	0.3
Ecuador	293.139	454	590	0.2
Guatemala	423.960	119	165	0.04
Mexico	2.586.287	4.374	5.911	0.23
Nicaragua	117.500	50	59	0.05
Panama	86.134	280	359	0.42
Paraguay	86.970	29	54	0.06
Peru	568.882	1.419	1.577	0.28
Rep Dominicana	159.532	51	88	0.06
Uruguay	48.200	367	448	0.93
Venezuela	615.132	76	271	0.04
